# Selective Reduction of Iron in High-Phosphorus Oolitic Ore from the Lisakovsk Deposit

**DOI:** 10.3390/ma17215271

**Published:** 2024-10-30

**Authors:** Bakyt Suleimen, Nurlybai Kosdauletov, Galymzhan Adilov, Pavel Gamov, Semen Salikhov, Yerbol Kuatbay, Talgat Zhuniskaliyev, Bauyrzhan Kelamanov, Almas Yerzhanov, Assylbek Abdirashit

**Affiliations:** 1 Department of Metallurgy and Materials Science, Karaganda Industrial University, Temirtau 101400, Kazakhstan; bakytsuleimen@mail.ru (B.S.); kosdauletovn@susu.ru (N.K.); adilovg@susu.ru (G.A.); ye.kuatbay@tttu.edu.kz (Y.K.); t.zhuniskaliyev@outlook.com (T.Z.); 2 Department of Pyrometallurgical and Foundry Technologies, South Ural State University, Lenin Prospekt 76, Chelyabinsk 454080, Russia; gamovpa@susu.ru (P.G.); salikhovsp@susu.ru (S.S.); 3 Department of Metallurgy and Mining, K. Zhubanov Aktobe Regional University, Aktobe 030000, Kazakhstan; kelamanovb84@gmail.com

**Keywords:** oolitic, high-phosphorus iron ore, selective reduction, solid carbon, carbon monoxide, hydrogen

## Abstract

Reduction of iron in high-phosphorus oolitic ore from the Lisakovsk deposit using solid carbon, carbon monoxide, and hydrogen. An X-ray phase analysis was used to determine the phase composition of the samples after reduction roasting. When reduced with carbon monoxide or hydrogen, α-iron appears in the samples, while phosphorus remains in the form of iron, calcium, and aluminum phosphates. After roasting with solid carbon, phosphorus is reduced from iron and calcium phosphates and migrates into the metal but remains in aluminum phosphate. A micro X-ray spectral analysis showed that at a temperature of 1000 °C and a holding time of 5 h, during reduction with solid carbon, the phosphorus content in the metallic phase reaches up to 7.1 at. %. When reduced with carbon monoxide under the same conditions, the metallic phase contains only iron, and phosphorus is found only in the oxide phase. When reduced with hydrogen at 800 °C, phosphorus is almost absent in the metallic phase, but at 900 °C, phosphorus is reduced and its content in the metallic phase reaches 2.1 at. %.

## 1. Introduction

Currently, there is a discussion among scientists about the prospects of transitioning metallurgy to hydrogen technologies, which could significantly reduce greenhouse gas emissions, specifically carbon dioxide. Such a breakthrough in metallurgy would positively impact the selective reduction of metals from ores, as hydrogen is a weaker reducing agent compared to solid carbon.

High-phosphorus iron ore receives significant attention due to the difficulty of efficiently separating iron and phosphorus. High-phosphorus iron ores represent more than 60% of the reserves in all iron ore deposits in the Republic of Kazakhstan [1,2]. Thus, in the near future, we will be compelled to develop and process iron ores with high phosphorus content.

During the processing of high-phosphorus ores in a blast furnace, phosphorus transitions into pig iron, necessitating additional dephosphorization in subsequent steel production processes. This leads to an increased lime consumption, larger slag volumes, and complicates the technological chain of steel production. Attention should also be drawn to the illogical nature of the traditional two-step process when processing such ores. In the first step (during the blast furnace process), the metal is enriched with phosphorus at the cost of additional coke, while in the second step (steelmaking), significant effort is required for dephosphorization. This contradiction highlights the need for new technological solutions that would prevent phosphorus from entering the metal at the first stage of processing phosphorus-bearing iron ores, thereby eliminating the dephosphorization challenge in the steelmaking stage [3,4].

In recent years, most research on the dephosphorization of such ores has focused on pyrometallurgical processes, with the addition of various dephosphorizing agents [5,6,7,8,9], or on hydrometallurgical and chemical beneficiation methods that involve treatment with alkaline, acidic, or saline solutions [10,11,12,13,14,15]. These methods have not been implemented industrially due to the use of scarce and expensive materials, prompting the search for new methods. There is also a biological leaching method, which is less costly and more environmentally friendly, but the complexity of the process (e.g., acquiring leaching bacteria) often leads to low production efficiency [16,17,18,19].

In our previous work, we conducted thermodynamic modeling using the ‘TERRA’ software suite to simulate a selective solid-phase reduction of iron and phosphorus in oolitic ore, depending on temperature (1000–1400 K) and the amount of carbon in the system. The results showed that, with a specific amount of carbon in the system and a corresponding CO/CO_2_ ratio in the gas phase, selective iron reduction without phosphorus reduction is possible even at a temperature of 1100 °C [20]. The experimental feasibility of selective iron reduction by carbon monoxide from the Ayat deposit was also confirmed [20,21]. Additionally, studies [22,23] demonstrated the possibility of selective iron reduction by hydrogen in iron-manganese ores and ilmenite concentrate.

This study explores the possibility of selectively reducing iron in high-phosphorus oolitic ores from the Lisakovsk deposit.

## 2. Materials and Methods

The starting material used was oolitic iron ore from the Lisakovsk deposit, which has reserves of 6.5 billion tons. The characteristic feature of these ores is primarily their high phosphorus content, up to 0.6%, and up to 3.0–3.5% alumina (Table 1). A positive quality of these ores is their low sulfur content (0.02%) compared to the Ayat ores [24].

**Figure 1:**
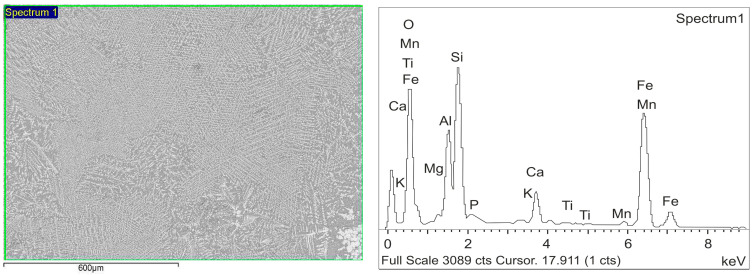
View of the raw ore after melting and quenching.

An electron microscopy analysis of the samples was conducted using a JEOL JSM-7001F scanning electron microscope with an OXFORD X-Max 80 energy-dispersive detector for elemental composition determination. The morphology and average chemical composition, determined after melting and quenching of the ore to homogenize the composition, are shown in Figure 1 and Table 2.

**Figure 2:**
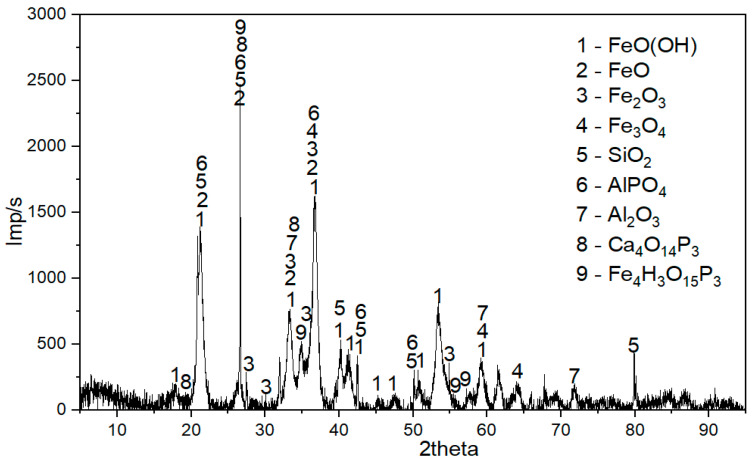
X-ray diffraction pattern of the raw oolitic iron ore.

Figure 2 shows the X-ray diffraction (XRD) analysis on a Rigaku Ultima IV diffractometer using the “Match 3.0” software, in which the primary phases of the raw ore are goethite FeO(OH), magnetite Fe_3_O_4_, and quartz SiO_2_. In addition to the main phases, compounds such as AlPO_4_, FePO_4_·2H_2_O, and CaHPO_4_·2H_2_O are also identified.

**Figure 3:**
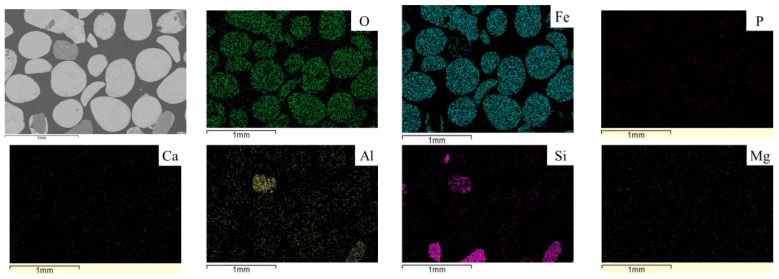
Elemental distribution maps in the raw oolitic iron ore.

In addition, the microstructure and element distribution were investigated using a scanning electron microscope JEOL JSM-7001F equipped with an energy-dispersive X-ray analyzer from Oxford Instruments, as shown in Figure 3. It is evident that some elements (O, Fe, Si, Al) can be visually correlated with the structural components presented in the image. The oolites are primarily composed of iron oxides. Other phases consist mainly of silicon and aluminum oxides. Additionally, complex phases containing multiple elements (Fe, Si, Al) are observed. Phosphorus, calcium, and magnesium are distributed almost uniformly across the image, making it nearly impossible to associate them with any specific phase.

**Figure 4:**
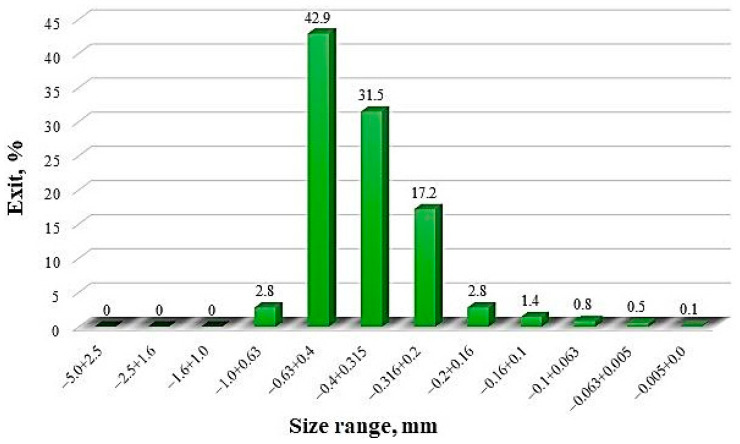
Granulometric composition of the original oolitic ore from the Lisakovsk deposit.

The results of the sieve analysis without crushing the original oolitic ore from the Lisakovsk deposit are shown in Figure 4.

As shown in Figure 4, the ore consists entirely of fine fractions less than 1 mm, with the majority of the material mass concentrated in the fractions of 0.63–0.4 mm (42.9%), 0.4–0.315 mm (31.5%), and 0.315–0.2 mm (17.2%).

**Figure 5:**
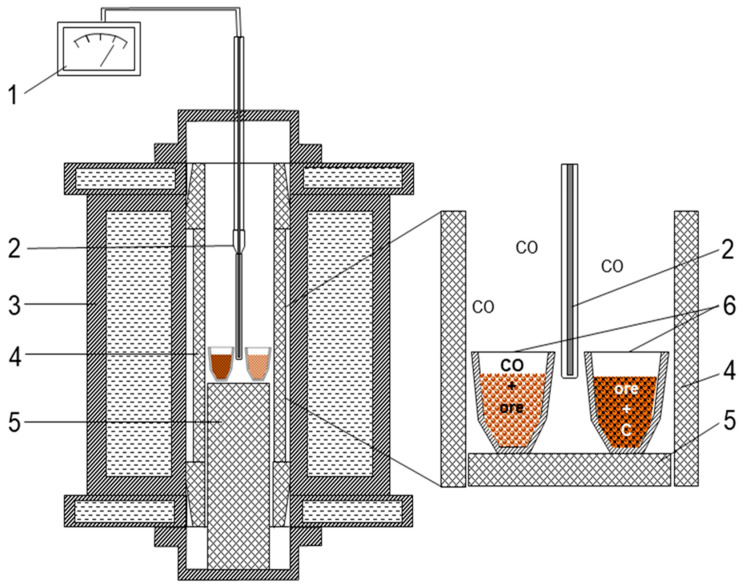
Experimental Setup: 1—multimeter, 2—thermocouple, 3—furnace body, 4—graphite heater, 5—graphite substrate, 6—material samples.

Experiments using solid carbon and carbon monoxide as reducers were conducted in a sealed resistance furnace with a graphite heater (Figure 5). Two corundum crucibles containing ore were placed in the furnace; in one crucible, the ore was mixed with ground graphite from used graphite electrodes. The furnace was covered with a lid, heated to 1000 °C, and held at this temperature for 5 h. In the closed furnace with a graphite heater, all oxygen is bound in carbon monoxide through the Boudouard reaction. Thus, the ore in one crucible was exposed only to a reducing atmosphere of CO, while in the other crucible, where additional graphite was added, it was also in contact with solid carbon. According to the calculation method [25], the equilibrium gas phase composition at 1000 K and 1 atm pressure in the furnace workspace with a graphite heater will be 28.06% CO, 4.03% CO₂, and 67.91% N₂. As the temperature increases, the CO content in the gas phase will rise, reaching 34.58% at 1273 K, while the CO₂ and N₂ contents will decrease to 0.07% and 65.35%, respectively. The temperature inside the reaction crucibles was monitored using a tungsten/tungsten-rhenium thermocouple WR5/20. After the holding period, the crucibles with samples were cooled along with the furnace to room temperature. The mixture of samples with graphite powder was dispersed to remove residual carbon, and samples were taken for micro-X-ray spectral and X-ray phase analyses, along with the original and CO-reduced samples. The chemical composition of the phases was determined using the micro-X-ray spectral method.

**Figure 6:**
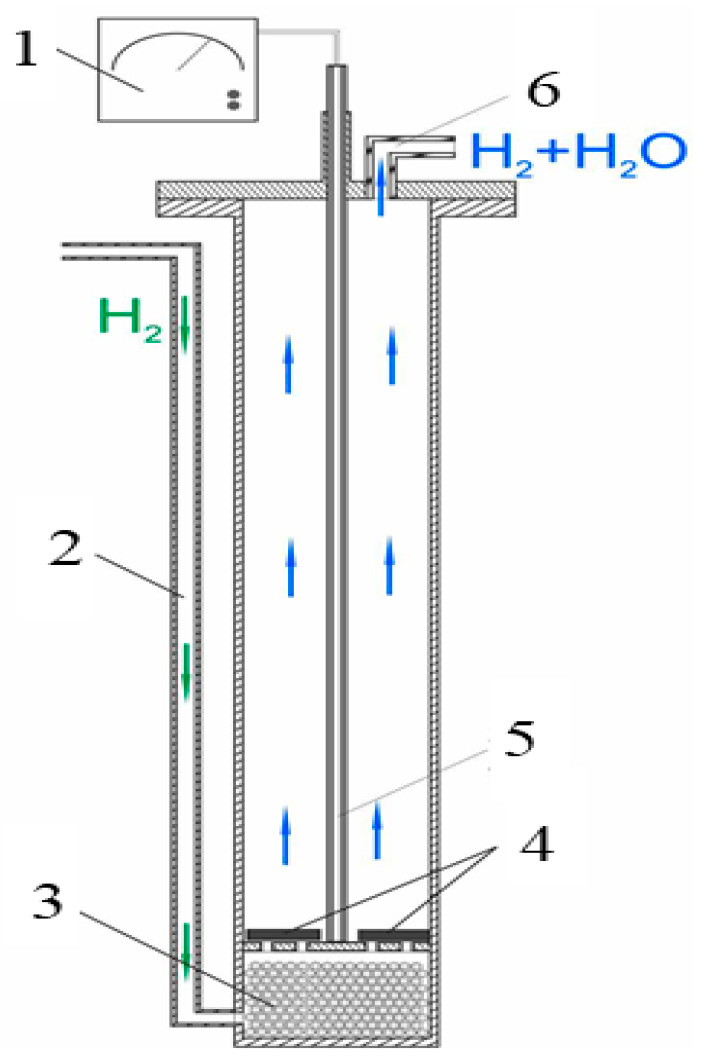
Reactor setup diagram: 1—multimeter; 2—hydrogen supply line; 3—corundum spheres for uniform gas distribution; 4—oolitic ore briquettes; 5—thermocouple; 6—exhaust gas line.

Hydrogen reduction experiments were conducted in an MM 6000 electric vertical furnace from RB Automazione, equipped with a frame, reaction chamber (Figure 6), and weighing system. Hydrogen (99.99%), according to GOST 3022-80, was used as the reducing gas, and high-purity argon (99.993%), according to GOST 10157-2016, was used as the inert gas. For the experiments, the initial oolitic iron ore was first ground into a fine powder with a particle size less than 0.4 mm and processed through an extruder to produce briquettes with a diameter of 8 mm and a length of 40 mm, following a previously established method [26]. The obtained briquettes (bricks) were weighed, placed in the furnace’s working zone, covered with a lid, and equipped with a thermocouple, a weighing system to record the sample mass throughout the experiment, and a gas supply tube. During the heating of the sample from room temperature to the desired temperature, the reactor was purged with argon at a flow rate of 0.5 L/min to remove air from the system. Once the target temperature was reached, the gas was switched from argon to hydrogen (5 L/min), and reduction was carried out at temperatures of 600, 700, 800, and 900 °C with a holding time of 20 min. After reduction, the hydrogen was switched back to argon (5 L/min), and the furnace heating was turned off. After cooling to room temperature, the samples were extracted, weighed, and the mass loss was calculated. Some of the samples, along with the original ones, were embedded in epoxy resin, polished, and examined using an electron microscope. The remaining samples were ground into fine powders and subjected to X-ray phase analysis.

## 3. Results and Discussion

**Figure 7:**
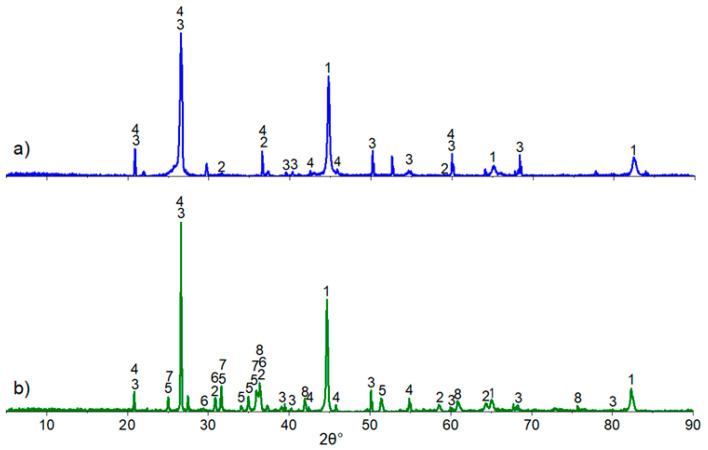
X-ray diffraction pattern of oolitic ore after reduction roasting with solid carbon; (**a**) carbon monoxide; (**b**) 1—Fe, 2—Fe_3_O_4_, 3—SiO_2_, 4—AlPO_4_, 5—Fe_2_SiO_4_, 6—Fe_2_P_2_O_7_, 7—CaP_2_O_6_, 8—FeO.

Figure 6 contains the results of the X-ray diffraction (XRD) analysis of samples after reduction roasting with solid carbon and carbon monoxide at 1000 °C for 5 h. In the diffraction pattern of the samples reduced with carbon monoxide CO (Figure 7b), there is a greater number of peaks and, consequently, a higher number of phases present compared to the samples roasted with solid carbon (Figure 7a).

According to the X-ray diffraction (XRD) results, both samples contain α-iron, magnetite Fe_3_O_4_, quartz SiO_2_, and berlinite AlPO_4_. In the samples reduced with carbon monoxide CO, iron is also observed in the form of fayalite Fe_2_SiO_4_ and wüstite FeO, and phosphorus is present in iron and calcium phosphates as FeP_2_O_7_ and CaP_2_O_6_ (Figure 7b). In contrast, the samples treated with solid carbon do not contain iron and calcium phosphates.

**Figure 8:**
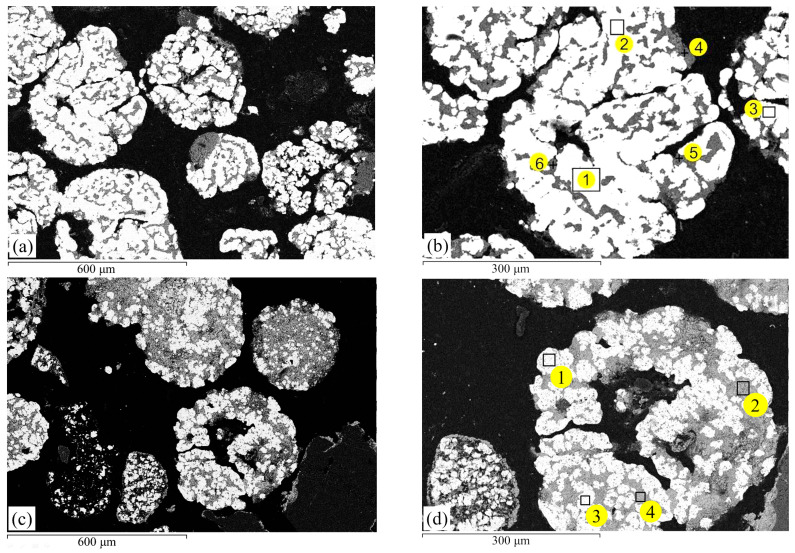
Polished sections of ore after reduction roasting at 1000 °C for 5 h with solid carbon (**a**,**b**) and carbon monoxide CO (**c**,**d**).

An examination of polished sections of metallized ores revealed that with both solid carbon and carbon monoxide CO, the metallic iron phase formed both on the surface and inside the ore particles (Figure 8). However, a reduction with solid carbon resulted in the formation of more distinctly defined, dense metallic structures (Figure 8a,b).

During the reduction of iron in oolitic ore with solid carbon at 1000 °C and a holding time of 5 h, the iron is almost completely reduced (with residual oxides containing 1.9–8.8%), and light areas are observed throughout the oolites (Figure 8a). In contrast, when reduced with carbon monoxide, the iron is not fully reduced; both light and dark areas of the oxide phase are present (Figure 8c), with iron remaining in the range of 16.5–21.7% (Table 3). In reduction with carbon monoxide (CO), the metallic phase contains only iron (spectra 1, 3, 5, Figure 8d), while phosphorus remains in the oxide phase. However, with reduction using solid carbon, the phosphorus content in the metallic phase reaches 1.5–7.1 at. % (spectra 1, 2, 3, Figure 8b).

According to the measurement results of mass loss for samples reduced with hydrogen (Table 4), it can be observed that with increasing temperature from 600 to 900 °C, the mass loss increases proportionally, from 21.9% to 34.0%. In our previous studies [27], an analysis of the mass change of the initial ore, with temperature, showed that during roasting in the air at a higher temperature (1200 °C), the total mass loss amounts to 14.5% due to moisture removal, dissociation of iron hydroxides, calcium and magnesium carbonates, and aluminum hydrophosphate. Considering these processes and mass losses, at a temperature of 600 °C, the mass loss is approximately 7.4%, indicating that the reduction process is occurring due to oxygen removal. After the experiment, the surface color of the samples changed from yellow to dark gray. Additionally, all samples become magnetic after the experiment.

**Figure 9:**
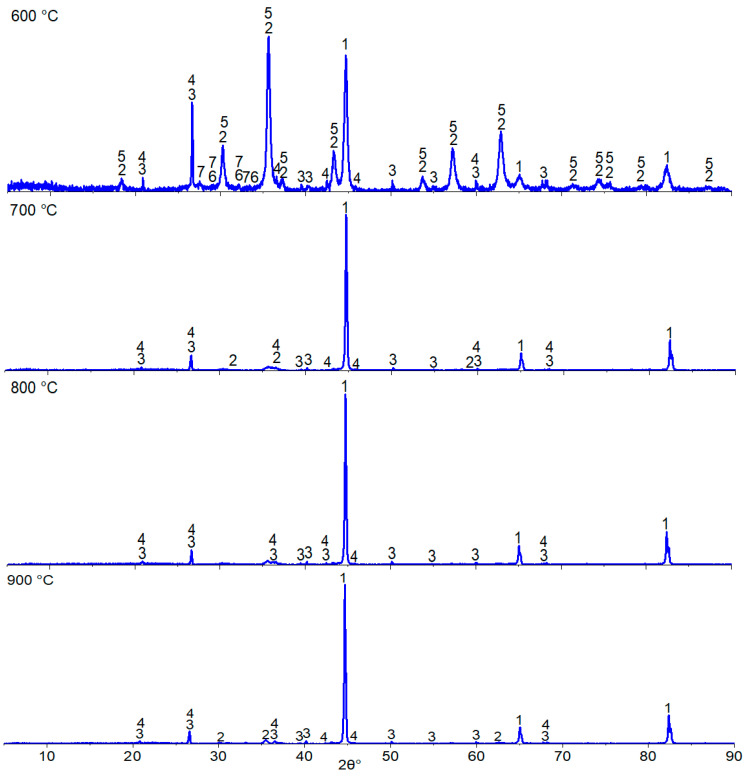
X-ray diffraction pattern of oolitic ore after reduction roasting with hydrogen: Phases: 1—α-Fe, 2—Fe_3_O_4_, 3—SiO_2_, 4—AlPO_4_, 5—Fe_2_SiO_4_, 6—Fe_2_P_2_O_7_, 7—Ca_2_P_2_O_7_.

Figure 9 shows the X-ray diffraction patterns of samples reduced at 600, 700, 800, and 900 °C. At 600 °C, reflections of the α-iron phase are observed, with their intensity increasing as the temperature rises to 900 °C, accompanied by a decrease in the intensity of magnetite and fayalite peaks. Additionally, all samples contain quartz (SiO_2_) and aluminum phosphate (AlPO_4_) phases. Phosphorus in the samples reduced at 600 °C is present as iron phosphates (Fe_2_P_2_O_7_) and calcium phosphates (Ca_2_P_2_O_7_). However, at reduction temperatures of 700 °C and above, iron and calcium phosphate phases are no longer observed. We believe that with a significant increase in the intensity of the metallic iron peaks, the intensity of other peaks, including those of calcium and iron phosphates, decreases, although this does not exclude their presence.

**Figure 10:**
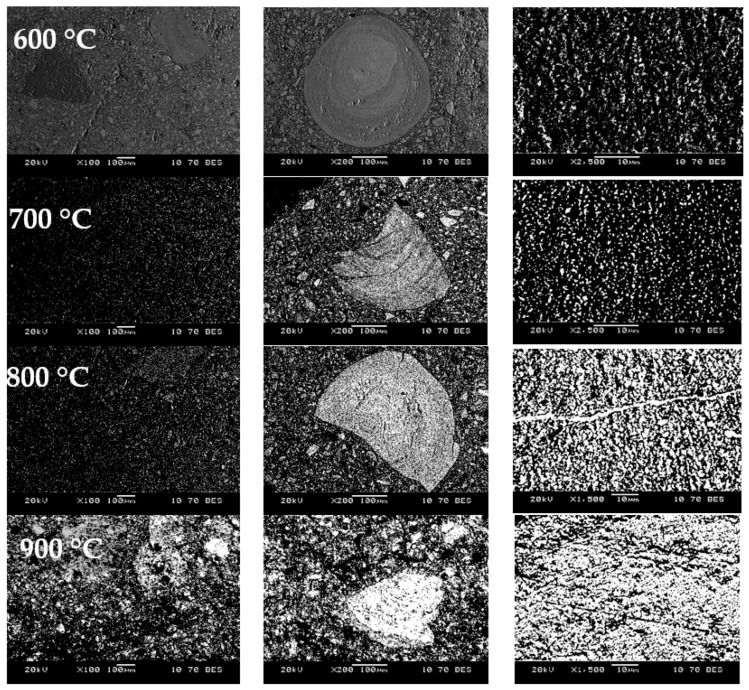
SEM of oolitic ore after reduction roasting with hydrogen.

Figure 10 shows the results of micro-X-ray spectral analysis of samples reduced with hydrogen at temperatures of 600, 700, 800, and 900 °C.

It should be noted that the amount of light (metallic) phases gradually increases with the rise in reduction temperature (Figure 10). In samples reduced at 900 °C, the quantity of light phases significantly exceeds the quantity of dark (oxide) phases. In contrast, in samples reduced at 600 °C, only dark phases are visible, with small light phases appearing only under high magnification (2500×).

**Figure 11:**
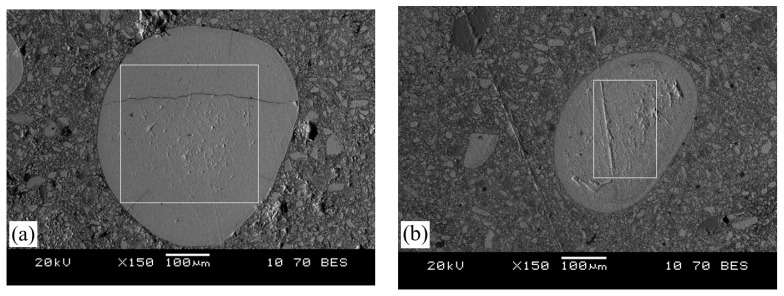
Analysis area of oolitic ore after reduction roasting with hydrogen at temperatures of 600 °C (**a**) and 700 °C (**b**).

In samples reduced with hydrogen at temperatures of 600 and 700 °C, it is practically impossible to quantitatively assess the composition of the metallic phases even under maximum magnification. Therefore, Figure 11 shows the phase area analysis of the oolite reduced with hydrogen, with the elemental composition provided in Table 5.

**Figure 12:**
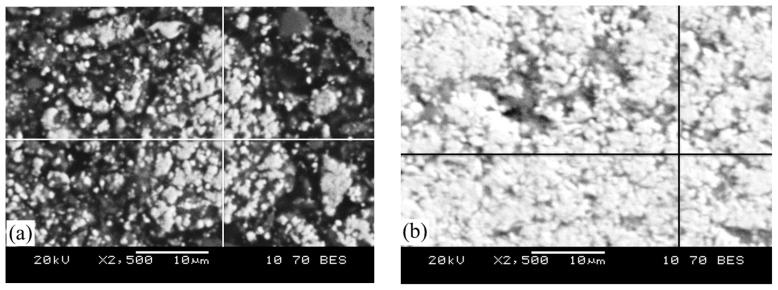
Composition of the metallic phase of oolitic ore after reduction roasting with hydrogen at temperatures of 800 °C (**a**) and 900 °C (**b**).

Elemental content of the metallic phase (at points) at high magnification (2500×) in the reduced samples at temperatures of 800 °C and 900 °C is presented in Table 6 and Figure 12.

As a result of hydrogen reduction at 800 °C, the metallic phase contains only iron. When the temperature is increased to 900 °C, phosphorus transitions into the metallic phase, reaching approximately 2.1 at. %.

The X-ray diffraction analysis results showed that in all metallized samples, goethite disappears, the α-iron phase appears, and the SiO₂ phase remains. In samples reduced with carbon monoxide or hydrogen, fayalite and magnetite phases appear. As the reduction temperature with hydrogen increases from 600–900 °C, the intensity of the peaks of these phases decreases while the peak of metallic iron increases. Phosphorus in samples reduced with carbon monoxide (CO) is present as CaP_2_O_6_, FeP_2_O_7_, and AlPO_4_ compounds, whereas in samples reduced with solid carbon, phosphorus remains only in the AlPO_4_ phase. Under these conditions, phosphorus is reduced from calcium and iron phosphates and transitions into the metal. During hydrogen reduction at 600 °C, phosphorus is present as calcium, iron, and aluminum phosphates, while at temperatures of 700 °C and above, phosphorus remains only in the AlPO₄ phase. These results are confirmed by electron microscope analysis after reduction roasting. In samples reduced with carbon monoxide at 1000 °C or hydrogen at temperatures of 600–800 °C, phosphorus is almost not reduced. However, with hydrogen reduction at 900 °C or in combination with solid carbon at 1000 °C, phosphorus is reduced and detected in the metallic phase by micro X-ray spectral analysis. This is due to solid carbon being a stronger reducer at these temperatures, capable of breaking down more stable phosphorus oxides and reducing both iron and phosphorus, while carbon monoxide is a weaker reducer and can only reduce iron but not phosphorus. Additionally, by controlling the temperature during hydrogen reduction, it is possible to provide thermodynamic and kinetic conditions for the selective reduction of iron and phosphorus. At lower temperatures, only iron is reduced as hydrogen lacks sufficient energy to reduce phosphorus from stable oxides. At higher temperatures, thermodynamic conditions become more favorable, reaction kinetics improve, and hydrogen reduces both iron and phosphorus.

Thus, the experimental results indicate that during gaseous reduction, specifically with CO or hydrogen, it is possible to selectively reduce only iron from oolitic ore while leaving phosphorus in the oxide phase.

## 4. Conclusions

At a temperature of 1000 °C with a 5 h holding time, carbon monoxide does not reduce phosphorus from iron and calcium phosphates, nor from aluminum phosphates. Under the same conditions, in contact with solid carbon, phosphorus is fully reduced and transitions into the metallic phase (up to 7.1 at. %) from calcium and iron phosphates, but it is not reduced from aluminum phosphate. Hydrogen reduction at 800 °C selectively reduces only iron, leaving phosphorus in the oxide phase. When the temperature is increased to 900 °C, phosphorus is reduced and transitions into the metal (2.1 at. %). Thus, the results confirm the feasibility of selectively reducing iron with carbon monoxide or hydrogen in high-phosphorus oolitic ores.

Solid-phase reduction can be performed using well-established roasting installations with a gaseous reductant. In this case, alternatives to carbon monoxide could be natural gas, hydrogen-enriched natural gas, or pure hydrogen. Using hydrogen instead of solid carbon and carbon monoxide would significantly reduce greenhouse gas emissions, as water vapor becomes the byproduct of the reduction. In the future, with the reduction of electricity costs, for example, through the development of nuclear energy, it might be possible to completely eliminate carbon as a reductant, replacing it with hydrogen, including hydrogen produced by the electrolysis of water.

## Figures and Tables

**Table 1:** Chemical composition of the oolite ore of the Lisakovsk deposit.

Chemical Composition (Per Dry Weight), %.								
Fe	Mn	P	S	SiO_2_	Al_2_O_3_	CaO	MgO	LOI
42.6	0.18	0.55	0.04	19.2	5.1	0.4	0.3	11.7

**Table 2:** Elemental Composition of the Raw Ore After Melting and Quenching.

Spectrum 1	O	Mg	Al	Si	P	K	Ca	Ti	Mn	Fe
at. %	66.3	0.7	7.3	11.6	0.5	0.1	1.8	0.0	0.3	11.2
mass. %	45.3	0.8	8.4	14.0	0.6	0.2	3.2	0.1	0.7	26.8

**Table 3:** Elemental content in the areas and points of ore analysis after reduction roasting at 1000 °C with a holding time of 5 h.

Analysis Area	Elemental Content, % (at.)							
	O	Mg	Al	Si	P	Ca	Mn	Fe
Spectrum 1 (Figure 8b)	–	–	–	–	1.8	–	–	98.2
Spectrum 2 (Figure 8b)	–	–	–	–	7.1	–	–	92.9
Spectrum 3 (Figure 8b)	–	–	–	–	1.5	–	–	98.5
Spectrum 4 (Figure 8b)	61.9	1.9	14.0	10.6	0.5	1.7	0.6	8.8
Spectrum 5 (Figure 8b)	64.4				0.4	1.5	0.8	3.2
Spectrum 6 (Figure 8b)	64.3				0.5	1.6	0.8	1.9
Spectrum 1 (Figure 8d)	–	–	–	–	0.0	–	–	100
Spectrum 2 (Figure 8d)	64.2				2.5	0.1	0.2	19.7
Spectrum 3 (Figure 8d)	–	–	–	–	0.0	–	–	100
Spectrum 4 (Figure 8d)	62.4				0.8	0.1	0.3	16.5
Spectrum 5 (Figure 8d)	–	–	–	–	0.0	–	–	100
Spectrum 6 (Figure 8d)	62.6	0.8	4.1	9.0	1.5	0.0	0.3	21.7

**Table 4:** Mass Loss of Metallized Samples.

Temperature, °C	Holding Time, min	Initial Sample Mass, g.	Sample Mass after Roasting, g.	Mass Loss, %
600	20	7473	5834	21.9
700	20	8768	6088	30.1
800	20	6458	4356	32.5
900	20	9834	6494	34.0

**Table 5:** Analysis of the lolite phase with elemental composition.

Reduction Temperature	Elemental Content, % (at.)				
	O	Al	Si	P	Fe
600 °C	20.8	8.0	4.3	2.0	64.9
700 °C	19.0	8.0	3.3	1.4	68.4

**Table 6:** Elemental composition in the analysis points of hydrogen-reduced products at temperatures of 800 and 900 °C.

Reduction Temperature	Elemental Content, % (at.)	
	P	Fe
800 °C	0.0	100
900 °C	2.1	97.9

## Data Availability

The data used to support the findings of this study are included within this article.
